# Utilization and farmers’ knowledge on pigeonpea diversity in Benin, West Africa

**DOI:** 10.1186/s13002-017-0164-9

**Published:** 2017-06-20

**Authors:** Mathieu Anatole Tele Ayenan, Agyemang Danquah, Léonard Essehou Ahoton, Kwadwo Ofori

**Affiliations:** 1 Department of Crop Science, School of Agriculture, College of Basic and Applied Sciences, University of Ghana, P. O. Box LG 44, Legon, Ghana; 2 Department of Crop Science, Faculty of Agronomic Sciences (FSA), University of Abomey- Calavi, 01 BP 526 Cotonou, Republic of Benin

**Keywords:** *Cajanus cajan*, Folk taxonomy, Four Cells Analysis, Medicinal uses; Symbology

## Abstract

**Background:**

Understanding factors driving farmers’ uses of crop genetic resources is a key component not only to design appropriate conservation strategies but also to promote sustainable production. However, in Benin, limited information is available on farmers’ knowledge related to pigeonpea uses and conservation. This study aimed at i) identifying and investigating the different uses of pigeonpea in relation with socio-cultural factors, namely age, gender, ethnic group and respondents’ residence, ii) assessing pigeonpea varieties richness at household level and iii) evaluating the extent and distribution of pigeonpea varieties.

**Methods:**

Three hundred and two farmers were surveyed using structured questionnaire. Direct observation, field visit and focus group discussion were carried out. Association between number of varieties maintained at household level and socio-cultural variables was tested. Mann-Whitney test was used to assess whether the number of varieties held by households headed by men and women were different. Distribution and extent of diversity was assessed through four cells analysis.

**Results:**

Farmers in Benin mainly grow pigeonpea for its grains for home consumption. Pigeonpea’s stem and leaves are used for medicinal purposes to treat malaria, dizziness, measles, and eye infection. The ethnic group and the locality of residence of farmers influenced on the use of pigeonpea for medicinal purposes (*P* < 0.01). There was no significant association (*P* > 0.05) between the number of varieties held by household and the age of the respondent, number of years of experience in pigeonpea cultivation, the size of household, number of family members engaged in agricultural activities and gender. Farmers used criteria including seed colors, seed size, plant height, maturity groups and cooking time to classify their varieties. Varieties with white seed coat color were the most grown while varieties with black, red or mottled seed coat color are being abandoned and deserve to be conserved.

**Conclusion:**

Knowledge on medicinal uses of pigeonpea is vertically transmitted within community and pigeonpea varieties maintenance at household level does not depend on socio-cultural factors. This study will contribute to raise awareness on the various utilization of pigeonpea. In addition, it provides the basis for designing conservation strategies of pigeonpea genetic resources.

## Background

Crop diversity contributes to increase food security, to alleviate poverty and to protect environment [1–4]. Cultural diversity in a given region is reflected through crop diversity, from which local population source their food and other goods and service to meet their ever-changing needs [1]. In recent years, the link between traditional knowledge and diversity of cultivated plants has drawn attention and a parallelism has been established between cultural diversity and biodiversity [5, 6]. Thus, various international agreements including Convention on Biological Diversity, International Treaty on Plant Genetic Resources for Food and Agriculture have emphasized on the contribution of indigenous knowledge in the maintenance of genetic diversity.

Traditional knowledge related to crop diversity includes different usages of crop species, their symbols for community, cultural value of the crop, preferences for cultivars, special recipes associated to the crop and genetic material exchange networks and management, songs and folks through which knowledge is transmitted [3, 7–9]. Threats on traditional knowledge related to diversity use and conservation are increasing [10]. Changes occurring in agricultural systems resulting from globalization, urbanization, agro-industrialization and intensification of agricultural systems have led to over reliance on production of few major crops and few elite cultivars [4, 10–12]. Consequently, there is a decline in production and diversity of so-called minor crops, important for food security in marginal areas, and the traditional knowledge associated to their conservation [13]. This rapid decline in diversity of neglected crops species and local knowledge systems related to their uses and management hampers agro-ecosystems resilience, reduces options for adaptation to changing biophysical conditions and limits the potential to develop improved varieties [10, 14, 15]. Understanding factors driving farmers’ uses of crop genetic resources is a key component to design conservation strategies and promote their cultivation and uses [12] notably for the minor or underutilized ones. In this perspective, traditional knowledge on uses and conservation of crop diversity has been documented for many crops (eg: [13, 16–20]).

In Benin, pigeonpea is a source of income and food security for rural household [20]. Despite its role in food security, pigeonpea falls in the category of genetic resources for food and agriculture found in farmers’ fields which are still to a large extent inadequately documented and managed [20, 21]. As such, drivers of uses and diversity maintenance of pigeonpea are still not well understood [20]. This is a threat for pigeonpea genetic resources since new varieties are being introduced in the growing areas (Authors’ personal observation). In addition, no systematic collection of pigeonpea genetic resources has been undertaken in the country yet. In order to avoid or limit the extent of genetic and knowledge erosion related production and uses of pigeonpea in the Benin, knowledge on uses and diversity of the crop that is currently held by farmers needs to be documented. Understanding the indigenous knowledge system in a minor pigeonpea growing country like Benin, is vital to advocate the importance of the crop as source of nutrients to cope with food insecurity and to introduce successfully new cultivars based on farmers ‘preferred traits [22]. Elsewhere, the investigation of the relationship between farmers’ knowledge and crop diversity revealed that various social, economic and cultural factors shaped the uses and the number of crop diversity maintained at community and household level [11, 23, 24]. Thus, crop varieties are considered as social objects [25]. We then hypothesized that socio-cultural factors influence uses of pigeonpea and the number of varieties maintained at household level. More specifically, we assumed that depending on socio-cultural factors such as age, gender, residence and ethnic group farmers may affect farmers’ knowledge and access to information regarding the uses of pigeonpea genetic resources. In addition, age, number of years of experience in pigeonpea cultivation, household size, and number of family members engaged in agricultural activities and gender may influence farmers’ variety preferences and seed movements which would drive the number of pigeonpea varieties and the kind of varieties farmers maintain at household level. Our study aimed at 1) identifying and investigating the different uses of pigeonpea in relation with socio-cultural factors including age, gender, residence and ethnic group, 2) assessing pigeonpea varieties richness at household level based folk taxonomy and 3) evaluating the extent and distribution of pigeonpea varieties based on farmers’ perception.

## Methods

### Study area

**Fig. 1:**
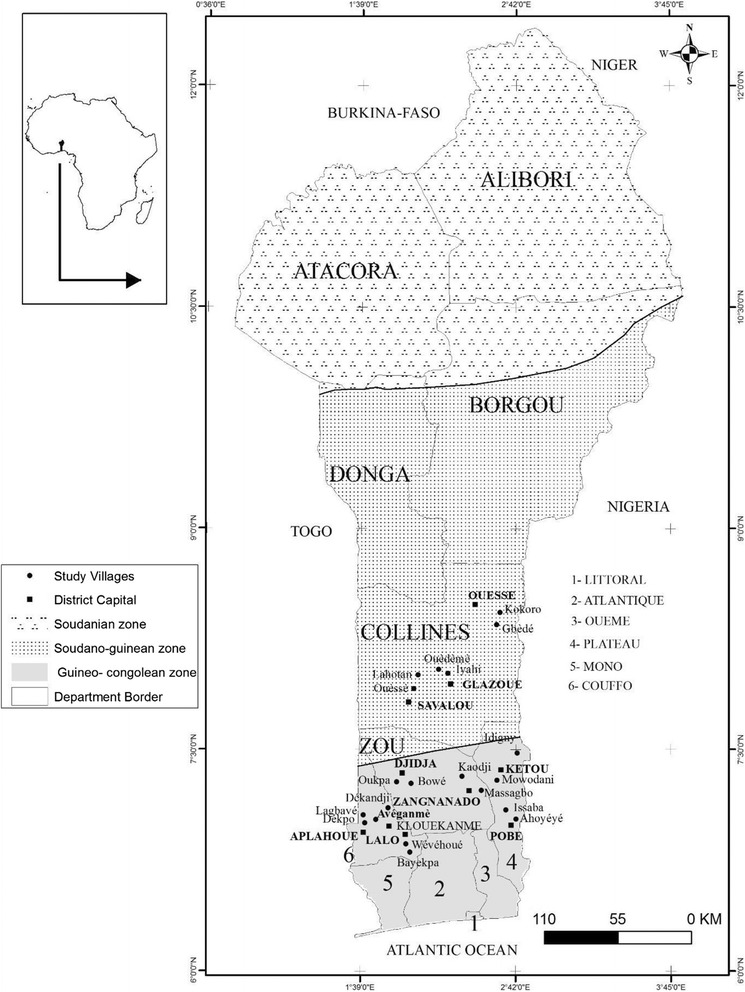
Map of the study area

The study was conducted in the major pigeonpea growing areas which were identified based on production figures obtained from the Ministry of Agriculture [26]. Data were collected in twenty villages (Fig. 1) spread over four Administrative Departments (Collines, Zou, Plateau and Couffo). In the study area, about 70% of the population lives in rural areas and agriculture is their major activity. Several ethnic groups and religions are encountered in these areas [27]. Maize, cassava, cowpea, yam, sorghum, rice and oil palm are the main cultivated crops [26] while pigeonpea is one of the minor crops cultivated by small farmers for subsistence and income generating [20, 28].

### Sampling technique and data collection

Respondents were selected using the snow-balling sampling technique as described by Biernacki [29]. Three hundred two (302) pigeonpea growers were reached in the growing areas [28]. Pigeonpea growers were 42 ± 1 (SD) years old on average with the majority of them (82%) being between 30 and 60 years old [28]. About 10% of the respondents were below 30 years old and 8% were above 60 years. Respondents who were less than 30 years old, between 30 and 60 years old and above 60 years old were considered as young, adult and old people, respectively [30]. Sixty-two percent of the pigeonpea growers were male. The average size of the household was six members among whom three persons on average were involved in agricultural activities [28].

A questionnaire was administered to sampled pigeonpea growers. Prior to the administration of the questionnaire, the objectives of the study were explained to farmers in local language and their informed consent was obtained. The questionnaire was divided into two main sections namely: 1) Uses of different parts of pigeonpea plants (seeds, stems, roots and leaves); 2) name and description of each grown variety, number of pigeonpea varieties held at the household level, desirable and undesirable traits of each variety as perceived by farmers.

In each village, a focus group was held with on average 12 pigeonpea growers including female and male to collect data related to the different varieties of pigeonpea grown and their characteristics. Data related to folk taxonomy, meaning of pigeonpea varieties in the various local languages encountered in the village, the plant’s role in ceremonies, or in symbolism were collected. For each cultivated variety in the village, the extent (perceptions of farmers on allocated area to its cultivation) and distribution (perception on the number of household growing the variety) were recorded. This information was used to assess the status of each variety in the village and to draw implications on its conservation.

### Data analysis

Descriptive statistics including frequencies and means were computed to describe quantitative data, namely percentage of respondents for each category of pigeonpea use (home consumption, commercialization and soil conservation) and number of pigeonpea varieties per location and households. We tested whether uses listed by respondents (frequency citation) were independent of their socio-cultural factors, namely age categories, ethnic group, region of residence and gender using Fisher Exact since frequency of some cells was less than 5 [31]. We investigated whether the number of varieties held at household level was correlated to age of the respondent, number of years of experience in pigeonpea cultivation, the size of household and the number of household members engaged in agricultural activities. We analyzed if the number of varieties held by households headed by men and women were different using the non-parametric Mann-Whitney test since the assumptions of normality were not met [31]. Analyses were done using R 3.2.2 [32].

Distribution and extent of cultivation of different varieties were assessed using the Four Cells Analysis [33]. Thus, in each village, farmers classified existing landraces into four groups using: i) varieties cultivated by many households on large areas; ii) varieties cultivated by many households on small areas; iii) varieties cultivated by few households on large areas, and iv) varieties cultivated by few households on small areas. The four cells analysis has the merit to identify varieties with high demand for livelihood, high demand on the market for quality traits and the rare varieties that should be considered for conservation [16, 17, 34, 35].

## Results

### Uses of pigeonpea

#### Home consumption and commercialization

**Fig. 2:**
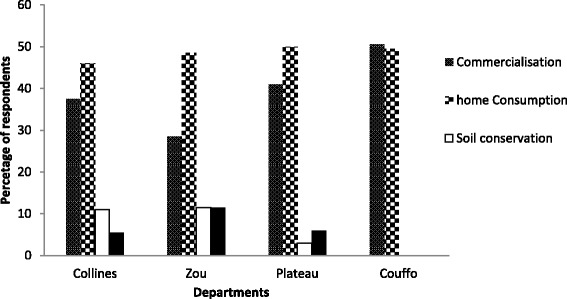
Reasons of cultivation of pigeonpea across the surveyed areas

All over the study area, 49% and 39% of pigeonpea farmers cited home consumption and commercialization respectively as the main reason driving their production of pigeonpea (Fig. 2). In the Department of Couffo, economic reason was the prevailing motive for pigeonpea production. Soil conservation and weed management was the third reason motivating the production of pigeonpea in Departments of Collines, Zou and Plateau (Fig. 2). Besides home consumption, commercialization was the second major purpose for growing pigeonpea in all the surveyed villages. In fact, farmers reported that the income from the sale of pigeonpea grains is used to pay labor at the onset of the cropping season (March–April). Thus, pigeonpea plays for the growers a strategic role in the production system. Dry pigeonpea grains are consumed in various forms. Seeds are boiled and mixed with “*gari*” (cassava derived product) or with maize flour and groundnut oil or palm oil. This form of consumption was the most popular across the surveyed area. In the Department of Plateau, whole dry seeds of pigeonpea are boiled with maize grains and the mixture is eaten with vegetable oil notably oil palm and groundnut oil. Other forms of consumption such as boiled pigeonpea and rice accompanied with sauce were reported. The important place of pigeonpea in home consumption is due to the fact that it is used to make up a shortage of cowpea, maize and other staple foods during lean season (May–June). According to respondents, this role is accounted for by its relatively long storage period, over 6 months.

#### Medicinal, firewood and fodder

The uses of pigeonpea plant parts (leaves and stems) depended on the ethnic group or locality of residence of farmers (*P* < 0.01) (Table 1). The leaves were used for feeding animal (goats) especially in the Department of Couffo mainly by Tchi. The use of pigeonpea leaves in folk medicine to cure various diseases was reported all over the study areas but it was predominant in the Departments of Zou and Plateau. Farmers used pigeonpea for medicinal, fodder or firewood regardless of their age and gender (*P* > 0.05) (Table 1). This finding suggests that knowledge related to pigeonpea for medicinal purposes is widely shared within a given community.

**Table 1:** Use frequency of pigeonpea across socio-demographic factors

Socio-demographic factors		Medicinal	Firewood	Fodder	Probability of Fisher Exact test
Age category	Youth	9	14	0	*P* = 0.1504
	Adult	91	183	6	
	Old	8	29	3	
Gender	Male	60	133	5	*P* = 0.8392
	Female	48	93	4	
Ethnic group	Adja	24	81	5	*P* < 0.01
	Fon	36	41	0	
	Holli	10	17	0	
	Tchi	1	16	3	
	Idaatcha	11	17	0	
	Nagot	12	30	0	
	Mahi	17	22	0	
Locality of origin	Couffo	24	96	8	*P* < 0.01
	Plateau	48	59	0	
	Zou	22	19	0	
	Collines	17	50	0	

Malaria was the most treated disease with pigeonpea and it was reported in all the surveyed villages, with some specificity in regard with the form of administration (Table 2). In regards to specificity in the uses of pigeonpea in folk medicine, the ethnic group Holli in Pobè and Ketou municipalities (South East Benin) used the filtrate obtained after triturating the leaves to treat fever, dizziness and eye infection while the use of leaves was reported in the treatment of measles by ethnic groups Idaatcha, Mahi, Fon and Nago in the Departments of Zou and Collines (Central Benin). The use of pigeonpea leaves in treatment of some ailments was locality-dependent (Table 2). This finding revealed that, depending on the locality of residence, farmers hold specific knowledge related to the medicinal uses of pigeonpea. In Wévéhoué village (Department of Couffo), pigeonpea is used in ritual ceremony. Dry pigeonpea seeds are boiled and eaten during twin’s naming ceremony. It is worth noting that the different medicinal uses were not associated with a specific pigeonpea variety.

**Table 2:** Medicinal uses of pigeonpea

Diseases	Forms of use	Form of administration or application	Villages
Malaria	- Triturate the leaves, filter and add lemon juice - Triturate leaves, and add either lemon or citronella leaves or both and then filter the mixture - decoction of pigeonpea leaves and acacia leaves	Drinking of the filtrate	All the villages
Ulcer	- Leaves decoction	Drinking of the decoction	Zagnanado (Kaodji, Massagbo)
Measles (children)	- Add water to triturated leaves - Leaves decoction	Use as bath water to treat children and/or drinking of the decoction	Ouesse (Kokoro), Djidja (Oukpa), Zagnanado (Kaodji, Massagbo)
Fever	- Triturate fresh leaves, add to water and filtrate - Triturate fresh leaves, filtrate and add to vine or fermented water	Use as bath water and/or drinking of the filtrate	Pobè (Issaba, Ahoyéyé)
Snake bite	Triturate fresh leaves	Apply the triturated leaves on the bite	Djidja (Oukpa, Bowe)
Eye infections	- Triturate fresh leaves and filter	Drop the filtrate in eyes	Pobè (Issaba, Ahoyéyé)
Dizziness	- Triturate fresh leaves and filter	Drink the filtrate	Pobè (Issaba), Kétou (Mowodani)

### Symbology

Pigeonpea production in Benin is linked to two key symbols. The most ancient went back to mid-nineteenth century (Story recorded during discussion group organized in March, 2015 in Klouékanmey), and it is related to the name Klouékanmey, a municipality, located in the Department of Couffo (South-West Benin). Klouékanmey in local language “*Adja*” means “Pigeonpea area”. The name was given based on the introduction and cultivation of the crop in this area, which has remained one of the major pigeonpea growing regions in Benin.

Since 2013, a yearly festival is organized in Pobè to celebrate pigeonpea. Thus, each Easter, Pobè people organize a festival to make a showcase of the different pigeonpea based meals and to promote the cultivation of this crop owed to its economic importance for local population. This event named *“Odun Otini”* in *Nago* that literally means “Celebration of Pigeonpea” offers a unique opportunity of reunion the member of this community (Story recorded during group discussion in Pobè in March, 2015). This emphasizes the role of pigeonpea in socio-cultural life of this community and its contribution to build social link.

### Folk taxonomy, varieties richness

#### Folk taxonomy

Farmers in the surveyed areas have local name for pigeonpea (Table 3). The meaning of local name of pigeonpea depended on the ethnic group. The existence of local name of pigeonpea showed the long history of cultivation of the crop and its cultural value in the growing areas.

**Table 3:** Local names given to pigeonpea and meaning in the different ethnic group

Ethnic groups	Name
Adja and Tchi	*Eklui*
Fon and Mahi	*Klouékoun*
Idaatcha	*Kolo*
Nago from Collines department	*Otili*
Holli and Nago from Plateau department	*Otini*

Varietal naming of pigeonpea based on farmers’ knowledge varied across surveyed area but the name of the varieties were either based on the seed color, or the origin of the variety (*Adja klui*: pigeonpea originated from Adja region), the perception of farmers on the variety (*Klui Gbali*: simple pigeonpea). Considering the grain color, the literally translated “white pigeonpea” variety was named *Otini founfoun*, *Kolo foufoun*, *Klouékoun wéwé* by Nago/Holli, Idatchaa and Mahi/Fon, respectively. Sixteen varieties were identified of which 5; 4; 4 and 3 varieties were recorded in the Departments of Couffo, Plateau, Zou and Collines, respectively. Varietal classification of pigeonpea by producers were based on 8 criteria including morphological (seed colors, seed size, plant height), physiological (maturity groups), agronomic (productivity, sensitivity to insects attack) and organoleptic and culinary (taste, cooking time).

#### Varieties richness

At household level, the number of pigeonpea varieties held by producers ranges from 1 to 3. Twenty eight percent (28%), 25% and 13% of the respondents in the Departments of Plateau, Zou and Collines cultivated two varieties, respectively while in the Department of Couffo each household grew one variety. The highest number of varieties (3) per household was reported in Zou and maintained by only one farmer. There was no significant association (*P* > 0.05, *n* = 302) between the number of varieties held by household and the age of the respondent, number of years of experience in pigeonpea cultivation, the size of household and number of family members engaged in agricultural activities. Similarly, there was no significant (*P* > 0.05) difference between the number of varieties held by men and women (Table 4). None of the socio-cultural factors investigated determined pigeonpea varieties richness at household level.

**Table 4:** Relationship between socio-demographic variables and number of pigeonpea varieties held at household level

		Age of respondent	Number of years of experience in pigeonpea cultivation	Size of household	Number of family members engaged in agricultural activities
Correlation test	Number of varieties	*P* = 0.179	*P* = 0.619	*P* = 0.4	*P* = 0.263
		*R* = −0.078	*R* = −0.029	*R* = −0.049	*R* = 0.065
Mann-Whitney test	Female	1 (median)			
	Male	1 (median)			
	W = 16,999.0 *P* = 0.9308				

### Distribution and extent of pigeonpea varieties

**Fig. 3:**
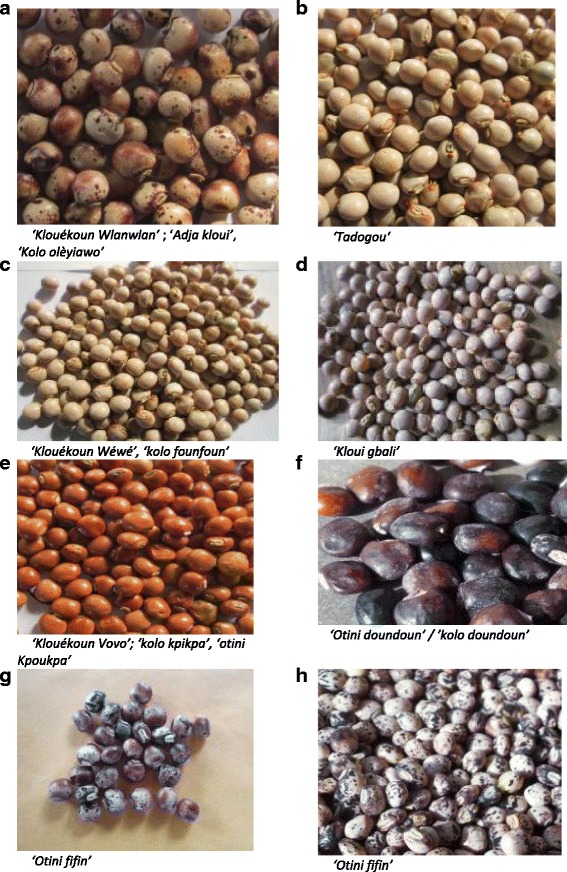
Common varieties of pigeonpea cultivated in Benin. **a** *Klouékoun Wlanwlan/Adja kloui/ Kolo olèyiawo;* **b** *Tadogou;* **c:** *Klouékoun Wéwé/ kolo founfoun;* **d** *Kloui gbali;* ***e*** *Klouékoun Vovo/kolo kpikpa/ otini Kpoukpa;* **f** *Otini doundoun / kolo doundoun*; **g** *Otini fifin;* **h** *Otini fifin*

The Four Cell Analysis showed that varieties with white primary seed color were the most grown not only in terms of allocated area but also in terms of number of households cultivating them. The varieties with red, spotted, black primary seed color were not preferred by consumers and their production tended to decline. As a result, the following varieties *Klouékoun vovo*, *Klouekoun wlanwlan*, *Klouékoun*, *Klui gbali*, *Adja Kloui*, *Otini kpoukpa*, *Otini fifin*, *Otini doudou*, *Kolo kpikpa* (Fig. 3 a, e, f, g and h) (Table 5) are likely to be totally abandoned by farmers because as previously mentioned, consumers do prefer the white seed varieties to them. Varieties like *Kloui gbali* grown in Dékandji, Wéwéhoué and klouékoun cultivated in Bayékpa were still being cultivated by many households in these villages because they were the only variety available for farmers. Furthermore, farmers reported the disappearance of some varieties like *Adja kloui* in Lagbavé, Dékandji and Avéganmè villages and *Gbahoun kléli* in Dekanji. The abandonment of their production was due to their undesirable traits such as long cooking time, small grains, late maturing, tall plant and low yield (especially for *Gbahoun kléli*). Recently pigeonpea cultivar named *Tagodou* (Fig. 3b) with big seeds, high yielding, sweet taste, medium plant height and short cooking time, has been introduced in this area and it is being adopted by farmers leading to the abandonment of the *Adja kloui* (Fig. 3a) and *Gbahoun kléli* variety. *Adja klui* (Fig. 3d) was being cultivated in some villages where it was the only one variety available. This finding suggests that farmers cultivate some pigeonpea landraces because they have limited access to improved varieties. Access to improved varieties could be a threat for the in-situ conservation of these landraces.

**Table 5:** Local names and characteristics of varieties

Varieties with in bracket the ethnic groups	Meaning of Names	Agro-morphologic traits	Other traits	Distribution and Extent
*Klouékoun wéwé* (Mahi, Fon)	White pigeonpea	White seed, late maturing, Tall plant	long conservation period, Long cooking time	**+ +**(Kaodji, Massagbo, Bowe, Oukpa)
*Klouékoun wéwé* (Mahi, Fon)	White pigeonpea	White seed, early maturing (2 harvest per year: 3 months after sowing and 9 to 10 for second harvest), High productivity	Short cooking time	**- -** (Bowe), not known by many pigeonpea growers yet
*Klouékoun vovo* (Mahi, Fon)	Red pigeonpea	Red seeds, late maturing	Less preferred by consumers because of the seed color	- - (Logozohè, Massagbo)
*Klouékoun wlanwlan* (Mahi, Fon)	Spotted pigeonpea	White seeds spotted with purple, late maturing (9–10 months)	Long conservation period, long cooking time, less preferred because of seed color, become black after cooking	- + (Kaodji, Massagbo, Bowe)
*Kpèdovinonwov*o (Mahi)		Early maturing (7 months), white seeds color with reddish spots, plant is very short		- - (Lagbavé)
*klouékoun* (Tchi)		Small seeds, late maturing (9–10 months), Tall plant, Medium productivity	Long cooking time	**+ +** (Bayékpa) Only one available cultivar in the village
*Kloui gbali* (Adja, Tchi)	Ordinary pigeonpea	Tall plant, small seeds, low productivity, late maturing (9–10 months)	Long cooking time	**+ +(**Dékandji, Wéwéhoué) Only one available cultivar in the village
*Adja Kloui* (Adja) Dekpo centre	Pigeonpea from adja area	Tall plant, small seeds, late maturing (9–10 months)	Long cooking time	- - (Dekpo-Centre)
*Carder klui* or *Tagoudou* (Adja)		Big seeds, medium height, late maturing (9 mois)	Taste good, high productivity, Short time for cooking,	**+ +** (Lagbavè, Aveganmè) Dekpo-centre
*Otini founfoun* (Holli, Nago)	White pigeonpea	White seeds color, late maturing (9 months), early maturing (6 months), tall plant,	Short time for cooking, taste good (sweet), high productivity, most preferred on the market, Susceptible to pests	**+ +(**Issaba, Ahoyéyé, Idigny); + − (Mowodani)
*Otini kpoukpa* (Holli, Nago)	Red pigeonpea	Red seeds color, late maturing (8 months), tall plant, long cooking time	Less preferred by consumers because of the seed color (Gbede), taste sweet (Idigny), seeds are highly sensitive to pests	**- -** (Issaba: recently introduced and has a lot of undesirable traits) **+ +(**Idigni, Mowodani: early maturing)
*Otini doudou* (Holli, Nago)	Black pigeonpea	Black seeds color, High productivity, long maturing	Long cooking time, highly sensitive to insects, taste sweet	--(idigny: undesirables traits)
*Otini fifin* (Nago)		White seeds spotted with black, tall plant, Very High productivity, late maturing (9 months)	Short cooking time,	--(Mowodani: less preferred on the market)
*Kolo Founfoun* (Idaatcha)	White pigeonpea	White seeds, late maturing, Tall plant	Taste sweet	++(Magoumi)
*Kolo kpikpa* (Idaatcha)	Red pigeonpea	Red seeds color, late maturing, Tall plant,		**- -** (Gbede)
*Kolo èko* (Idaatcha)		Red seeds color; late maturing	Not preferred by consumers	No more cultivated in the Magoumi village (Municipality of Glazoué)

**NB:** ++ varieties cultivated by many households on large plots

- - variety cultivated by few household on small plots.

- + variety cultivated by few households on large plots.

Early or late maturity in the distribution and extent of varieties were used in conformity with farmers’ perception

## Discussion

### Farmers’ knowledge on pigeonpea uses

Pigeonpea is used in various ways. Boiled whole dry seeds were the main form of consumption. Pigeonpea seed boiling was shown to be important in reducing anti-nutritional factors notably trypsin and increasing digestibility of protein and carbohydrate [36]. The important place of pigeonpea in home consumption is due to the fact that it is used to make up for the shortage of cowpea, maize and other staple foods during lean season (May–June). The use of immature seeds as vegetable was not reported in Benin conversely to what is observed in Eastern African countries and other parts of the world [37–39]. Besides home consumption, commercialization was the second major purpose for growing pigeonpea in all the surveyed villages. Income generated by the sale of pigeonpea grains is used to pay labor at the onset of the cropping season (March–April). This finding underscores the strategic role played by pigeonpea in the production system. Local populations use dry stems and leaves for several purposes (food, firewood, fodder, medicines). This multi-purpose characteristic of pigeonpea, reported across its growing areas worldwide [22], is particularly due to the perennial nature of most of the genotypes [40].

The use of pigeonpea leaves in treatments of some diseases (dizziness, snake bite) was ethnic group and locality-dependent. This finding showed that pigeonpea farmers in Benin do not have the same knowledge on the use of pigeonpea and specific knowledge related to the plant uses might be kept and transmitted within communities in some areas as a result of vertical knowledge transmission [3, 25]. However, male or female and youths or adults do not hold exclusive knowledge related to the use of pigeonpea for medicinal purposes. This may be a result of horizontal transmission of information among gender and age categories regarding pigeonpea uses [3, 25]. The use of pigeonpea leaves to treat various diseases such as malaria was reported by farmers in many countries and substantiated by pharmacological or antiviral tests [41–43]. These observations give a scientific support to indigenous knowledge in the identification of plants for treating diseases. The treatment of eye infections and vertigo reported by farmers is confirmed by previous studies [22]. Use of pigeonpea leaves to treat snake bite has not been reported elsewhere. Further researches on the biologically active or the properties of pigeon pea responsible for curing snake bite are needed.

No specific pigeonpea variety was associated with folk medicines conversely to previous findings on *Macrotyloma geocarpum* (Harms) Maréchal & Baudet) [18, 44] in Benin and on rice [23] in Nepal, showing that some landraces were specifically used for medicinal purposes. These results suggest that usage of crop varieties in folk medicine depends on the crop species and the cultural background of local communities. Furthermore, the fact that no particular cultural and religious use was associated to a given variety may be a threat for the conservation of grown varieties when new varieties are introduced in the farming systems. In fact, traditional values associated with varieties increase their chance of survival in farming systems [23, 45].

### Varieties richness, folk taxonomy, and conservation status of pigeonpea varieties

Assessing the relationship between socio-cultural factors and varieties richness at household level, no significant association was found between the number of varieties held by household and the age of the respondent, number of years of experience in pigeonpea cultivation, the size of household and number of family members engaged in agricultural activities. This finding does not substantiate our hypothesis and this could be due to the fact that the majority of farmers maintained and cultivated one variety at household level (very low varieties richness). In addition, even though pigeonpea plays an important role in food security, especially during lean season, the crop is not considered by farmers as a priority crop and they do not apply any external inputs and its cultivation does not need special skills. Thus, access to knowledge and resources, which are determined by social cultural and economic factors [3], may not have affected pigeonpea varieties richness. The absence of significant association between gender and diversity maintained at household level agreed with previous finding of Cromwell and Oosterhout [46]. However, Rana et al. [23] reported that male rice growers maintained more rice varieties, while Prain and Piniero [47] found that female farmers contribute more to on farm crop diversity management. All these results suggest that socio-cultural contribution to crop diversity maintenance and management is context-based.

Use of morphological, physiological, agronomic and organoleptic traits by farmers to discriminate their varieties was reported in various studies [19, 48]. Seed color, maturing groups and plant height were the predominant criteria used by farmers in the surveyed regions to classify and identify pigeonpea varieties. Manyasa et al. [39] reported seed size and maturity as the most important criteria used by Ugandan pigeonpea producers to discriminate their varieties while in this study seed color and maturity were the predominant criteria. In fact, farmers use various phenotypic characters to distinguish their varieties and these criteria vary across communities [3]. In this study, eight characters, fewer as compared with internationally described descriptors for pigeonpea, were used by farmers to distinguish the varieties. These criteria may not be enough to effectively differentiate varieties. However, beyond agronomic and morphological traits, farmers used organoleptic and culinary characters, which are not included in the descriptors. Some of the traits (taste of boiled grains) were based on the farmers and consumers preferred straits and they should be considered as breeding objectives in pigeonpea varietal development.

However, varietal taxonomy adopted by pigeonpea farmers depends on ethnic group and farmers’ location which may lead to some inconsistency. Therefore, one variety in farmers taxonomy may actually correspond to many varieties (one to multiple) and varieties differently named by farmers may in reality tally to one (multiple to one) [16, 19, 48, 49]. Such a situation may contribute to under or over- estimate the diversity within a species. To elucidate the situation, use of various descriptors through agro-morphological characterization back up with molecular tools are recommended [35, 50]. However, high consistency between farmers’ varietal classification and molecular markers was reported [11, 51], advising that assessing crop diversity based on folk taxonomy is still to some extent reliable.

In regard to varieties conservation status, varieties with white or cream primary seed color were the most grown not only in terms of allocated area but also in terms of the number of households cultivating. These varieties are not threatened. However, varieties with red, spotted, black primary seed color are not preferred by consumers and their production tends to decline. This observation substantiated findings in similar studies showing the abandonment of varieties farmers found less desirable [11, 37, 52, 53]. The abandonment of less preferred varieties could be explained by the fact that farmers’ preferences lie in their desire to adapt to their biophysical environment, cropping systems [12, 53] and meet consumers preferences (eg: preference to white seed color) [37, 52, 54]. Despite some undesirable traits mentioned by farmers as reason for their abandonment, these varieties may have some traits that can be exploited in breeding programs for further adaptation. In order to avoid the loss of those varieties, strategies for on farm conservation should be designed. In addition, seed exchange between farmers in the various growing areas should be encouraged with the support of extension agents. In fact, this action lies in the fact that varieties with less preference in some regions may be preferred in others. Furthermore, for ex situ conservation and exploitation of useful traits in Benin pigeonpea germplasm, collection and characterization of the cultivated pigeonpea varieties are advocated.

## Conclusion

Our study revealed that apart from the use of grain, farmers make use of pigeonpea in various ways depending on their ethnic groups and locality. The secondary uses of the crop are rarely mentioned by farmers as variety preference criteria but they can be of importance in the maintenance of plant genetic resource and determine the success of varietal introduction in a given community. Farmers showed a deep knowledge of their grown varieties through folk taxonomy. Medicinal uses of pigeonpea to treat ailments such as dizziness, snake bite, measles are determined by farmers’ location and ethnic group. No socio-cultural factor was found to determine maintenance of varieties at household level. Some of the varieties are being abandoned for their undesirable traits and they need to be considered under specific conservation strategy to avoid loss that may hamper future improvement of the crop. We proposed an extensive collection of Beninese pigeonpea genetic resource for further characterization and identification of useful traits to be exploited in the crop improvement programs.

## Abbreviations

INSAE: Institut National de la Statistique et de l’Analyse Economique
